# Does Social Support Affect the Health of the Elderly in Rural China? A Meta-Analysis Approach

**DOI:** 10.3390/ijerph16183471

**Published:** 2019-09-18

**Authors:** Natuya Zhuori, Yu Cai, Yan Yan, Yu Cui, Minjuan Zhao

**Affiliations:** College of Economics and Management, Northwest Agriculture and Forestry University, Yangling 712100, China; tuya@nwsuaf.edu.cn (N.Z.); caiyu1785@nwafu.edu.cn (Y.C.); yyan@nwafu.edu.cn (Y.Y.); cui2018060454@163.com (Y.C.)

**Keywords:** health of the elderly in rural areas, social support, influencing factors, meta-analysis, China

## Abstract

As the trend of aging in rural China has intensified, research on the factors affecting the health of the elderly in rural areas has become a hot issue. However, the conclusions of existing studies are inconsistent and even contradictory, making it difficult to form constructive policies with practical value. To explore the reasons for the inconsistent conclusions drawn by relevant research, in this paper we constructed a meta-regression database based on 65 pieces of relevant literature published in the past 25 years. For more valid samples to reduce publication bias, we also set the statistical significance of social support to the health of the elderly in rural areas as a dependent variable. Finally, combined with multi-dimensional social support and its implications for the health of the elderly, meta-regression analysis was carried out on the results of 171 empirical studies. The results show that (1) subjective support rather than objective support can have a significant impact on the health of the elderly in rural areas, and there is no significant difference between other dimensions of social support and objective support; (2) the health status of the elderly in rural areas in samples involving western regions is more sensitive to social support than that in samples not involving the western regions; (3) among the elderly in rural areas, social support for the older male elderly is more likely to improve their health than that for the younger female elderly; and (4) besides this, both data sources and econometric models greatly affect the heterogeneity of the effect of social support on the health of the elderly in rural areas, but neither the published year nor the journal is significant. Finally, relevant policies and follow-up studies on the impact of social support on the health of the elderly in rural areas are discussed.

## 1. Introduction

Next to the new economic normal, aging of the population has become another “new normal” in the development of the social population in China [1]. Influenced by the urbanization process, the rural population will suffer more severe aging than the urban population in China over a long period of time [2], and moreover, health issues of the elderly in rural areas will become increasingly prominent. The *Blue Book of Aging* points out that “Now, the quality of life of the elderly in China is not optimistic, and only about 30% of the elderly are in good health. The younger, highly educated male elderly in urban areas who have a spouse and don’t live alone are in better health condition [3]”. The impact of social support on the health of the elderly in rural areas has long been a topic of wide concern in social science and medical circles, especially in recent years, the related research has grown rapidly year by year [4,5]. However, existing studies usually come to different conclusions [6,7].

Social support includes objective, visible, or practical support, such as direct material aid or the existence of and participation in social networks and groups involving family, but it also includes subjective, experiential, or emotional support, such as the emotional experience and satisfaction degree of individuals being respected, supported, and understood [8]. In the existing literature, social support is defined from four dimensions, namely, subjective support, objective support, support availability, and their combination [9,10,11]. In studies, the health of the elderly is commonly defined from three dimensions, namely, physical health, mental health, and health self-assessment [12,13,14,15]. First of all, inconsistent conclusions have been drawn in studies of social support on a single dimension of health. For example, Zheng et al. discovered that objective support exerted a significant impact on the health self-assessment of the elderly [16], while Li et al. drew the opposite conclusion, believing that objective support cannot affect health self-assessment in the elderly [17]. Second, studies of a single form of social support on the health of the elderly have also come to different conclusions. With the effects of children’s care on the health of the elderly as an example, most studies have proved that children’s daily care helps the elderly to live better independently, meaning that the social support provided by children positively improves the health status of the elderly [5,18,19,20,21]. However, many studies also hold that the social support provided by children has little effect on the health of the elderly [22], and that social support provided by children is prone to making the elderly dependent. This is not conducive to improvement in the elderly’s ability to live independently, further resulting in an adverse effect on their health [16,23,24] and an increase in their probability of disease [25].

The quite different and even contradictory conclusions of research on the same topic affect the reliability of related studies, making it difficult to value their policy implications and suggestions and harming the theoretical and practical development of the health service security system for China’s elderly. Furthermore, the quite different and even contradictory conclusions of research in the same field are unlikely to give a clear future research direction and may even cause relevant researchers to have difficulty choosing or to make a biased choice, further resulting in research bias. Thus, it is necessary to explore the causes of these inconsistent research conclusions and make clear how social support affects the health of the elderly from different dimensions, thus providing reliable references for relevant studies and policies.

Meta-analysis is a multivariate regression method for literature [26] which is used to explore the significant differences between regression estimates or between their transformation forms (e.g., partial correlation, elasticity) and the variability between modeled values and real values [27,28] and to reveal the causes of inconsistent results through effect size, independent variable selection, and estimation analysis [29]. With various factors such as study geography, time, dependent variable measurement, and model form taken into account, meta-analysis is helpful in distinguishing biases and determining the accuracy of empirical research results [25,29].

To our knowledge, meta-analysis has been used only by Ge et al. to study the impact of social support on the health of the elderly in one piece of literature [30], in which they made a constructive attempt to explore the effects of social support on the health of the elderly and the relationship between them. However, since there were only four pieces of sample literature for study, it was difficult to eliminate the deviation of research results caused by accidental factors [26]. Moreover, the author determined that three of them were of “low quality” through Methodological Quality Assessment [30], which may have affected the validity of research results. In addition, this study, published in a medical journal, focused on the synthesis of research results rather than addressing the problems causing differences in research results, so it was unable to meet research needs in the field of economics with a social nature [28].

In the meta-analysis of influencing factors, the *t* value or its transformation value (such as Hedges’ *d* [31] or the partial correlation coefficient [32]) is commonly used to represent the effect size of studies. However, research literature always indicates and explains the significance of research results instead of the specific *t* value, so relevant samples are selectively excluded, aggravating the prevalent phenomenon of publication bias [28]. In this paper, the method of Stanley et al. was used to view “significance” as the effect size of a study and to include more effective samples [26]; this can weaken the deviation caused by publication bias to some extent.

The purpose of the study is to explore the causes of inconsistent conclusions in different studies concerning the impact of social support on the health of the elderly in rural areas, and further make clear how social support affects the health of the elderly from different dimensions by utilizing meta-analysis, thus providing reliable references for relevant studies and policies. Besides this, with significance as the effect size, this paper effectively increases the samples available for meta-regression. The later structure is as follows. Firstly, a meta-analysis database is established based on searching and coding of existing literature. Secondly, a meta-regression model is established with variables such as data type, measurement of dependent variables, model form, and socio-economic features taken into account. Lastly, the causes of inconsistent research conclusions are discussed according to the regression results to evaluate the model and provide predictions.

## 2. Data Search and Management

### 2.1. Sources of Data

**Figure 1:**
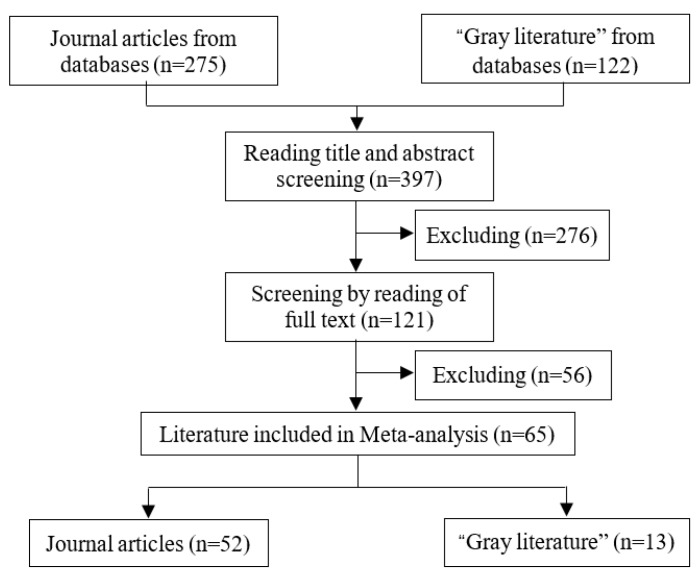
Literature screening processes and results.

On 8 August 2018, combinations of key words such as “health”, “old people” (or “aged” or “elderly”), and “support” (or “assistance”) were searched through the databases of China National Knowledge Infrastructure (CNKI) and Web of Science. To guarantee the quality of literature, the source category of journal articles in CNKI was limited to core journals. With the exception of literature with repeated contents and lacking specific empirical studies, a total of 275 journal papers, 102 academic papers, and 20 conference papers was initially searched (Figure 1).

### 2.2. Data Screening and Analysis

To establish a meta-analysis database, the following criteria were adopted for literature screening: (1) the target group of research involves the elderly in rural areas; (2) the topic of research involves the health of the elderly; (3) the empirical analysis involves an econometric estimation model; (4) the dependent variable of the econometric model represents the health of the elderly, and the independent variable includes at least one type of variable that represents social support; (5) all the key information of this paper is present (or a part of the information can be obtained through other channels); (6) it is written in Chinese or English. Both published and unpublished literature were included. The published literature mainly came from journals, while reports of work units, academic reports, and master’s or doctoral theses were classified as “gray” literature [33]. As a result, a total of 65 pieces of literature (52 pieces of journal literature and 13 pieces of “gray” literature) was finally selected, and 171 estimated results were obtained and included in the meta-analysis database for the purpose of later meta-regression analysis.

The literature contained in the meta-analysis database is set out in Table 1. Due to the differences in authors, fields, and journals, estimate indices had different forms, reflected in *p* values and *t* values representing degrees of significance. Of the literature, 52.31% clearly indicated the specific *p* value or *t* value, and 47.69% of the literature gave only a range of such values, meaning that the number of effective samples increased by 91.18% because we used significance as the effect size.

## 3. Modeling and Variable Selection

In general, literature, especially non-peer-reviewed and unpublished papers, tends to only report the significance of results and the information like *t*-value sometimes is not provided. Using the method recommended by Stanley and Doucouliagos, we shifted the focus of the meta-analysis from the analysis of effect size to the exploration of influences, namely, the statistical significance [26]. In this way, more literature is expected to be incorporated into meta-analysis and thus the selection bias of samples can be effectively reduced, despite loss of some information of samples. In this paper, the dependent variable was set as the statistical significance (at a 10% confidence level) of the impact of social support on the health of the elderly in rural areas in the study results.

In terms of the selection of independent variables, we further expanded the traditional basic form of meta-regression [28] to specifically include research object features, socio-economic features, model method features, and publication features (as shown in Table 2).

(1) Research object features: This involved the selection of two variables, namely, social support and elderly health in the literature. With reference to the Social Support Rating Scale developed by Xiao Shuiyuan, existing studies divided social support into three dimensions, i.e., subjective support, objective support, and support availability [41]. Most studies focused on analyzing how social support affects the health of the elderly from a single dimension [42,43,44,45,46], and some studies regarded social support as a whole for analysis [7,11,47,48]. In this paper, three groups of virtual variables were adopted to represent the type of social support, and objective support was viewed as a control group to figure out the differences in impact between objective support and other types of social support on the health of the elderly. The health of the elderly is commonly represented by two dimensions, i.e., physical health and mental health [49,50,51,52,53,54] or by self-assessment of physical health and mental health [36,45,55,56,57]. In this paper, two groups of virtual variables were adopted to represent the type of health of the elderly, and mental health was viewed as a control group to figure out the differences in impact between mental health and the other two health types on the health of the elderly.

(2) Socio-economic features: In terms of region, the meta-analysis database showed that socio-economic features are different in the eastern, central, and western regions. In terms of personal traits of the elderly, household registration, gender, age, education level, and marital status of the respondents are commonly taken into consideration in the existing literature [12,14,36,40,58,59,60,61,62]. In this paper, the proportion of respondents with rural household registration, proportion of male respondents, age, education level, marital status, and location (mainly in the western region where compared with other regions, the development of western China has lagged far behind and the elderly people living there thus have fewer endowments of social support, especially in rural areas, and we assume that as an input conforming to the law of diminishing marginal utility, social support can play a more important role in the health of the elderly in rural areas of western China) were selected as explanatory variables to be incorporated into the model.

(3) Model data features: The most obvious classification in samples is the model dependent variables being divided into binary variables (i.e., binary model) and non-binary variables. A binary model focuses on the determinants of the probability of an outcome, so that its dependent variables are two mutually exclusive discrete variables; this distorts the original variables’ data features to some extent. Consequently, binary models and non-binary models come to different estimated results [63]. The sources of data in samples were divided into open databases and other databases. Unlike other databases that are collected by individual researchers or institutes, open databases are sponsored and collected by national departments and can be used by anyone who applies. In this paper, two dummy variables, i.e., a binary model and open data, were established to determine whether model selection and data source affect the significance of results.

(4) Publication features: The published year reflects differences over time in research [28,64]. Publication bias has been a recognized issue in meta-analysis [65]. To reveal the possible effects of publication type on the significance of estimates, published year and publication type were used to represent publication features. (As mentioned above, it is impossible to figure out the complete estimation standard error of samples due to data limitations, so publication bias cannot be tested by funnel plot [66].)

Since the dependent variable is a binary variable, a Logit model was established for meta-regression analysis.

The significance of the impact of social support on the health of the elderly in rural areas is expressed by Sig.

(1) $$Sig=\{\begin{array}{ll} 1 & \mathrm{If}\;\mathrm{the}\;\mathrm{probability}\;\mathrm{is}\;p\\ 0 & \mathrm{If}\;\mathrm{the}\;\mathrm{probability}\;\mathrm{is}\;1-p \end{array}$$

The Logit model can be expressed as

(2) $$p=\Lambda (X^{\prime }\beta )=\frac{e^{X^{\prime }\beta }}{1+e^{X^{\prime }\beta }}$$

where Λ(⋅) is the cumulative distribution function of the logical distribution. Its probability density function is Λ(z) = $e^{z}/\left(1+e^{z}\right)$ = $1/(1+e^{-z})$, where X refers to a regression vector and β refers to a K×1 parameter vector. $X^{\prime }\beta$ can be specifically expressed as

(3) $$X^{\prime }\beta =\alpha +\beta_{o}X_{oi}+\beta_{s}X_{si}+\beta_{m}X_{mi}+\beta_{p}X_{pi}+u_{i}$$

where α is a constant vector and $\beta_{o}$, $\beta_{s}$, $\beta_{m}$, and $\beta_{p}$ refer to the parameter vectors of research object features $X_{oi}$, social-economic features $X_{si}$, model data features $X_{mi}$, and publication features $X_{pi}$, respectively. $u_{i}$ refers to a residual vector.

## 4. Results and Analysis of the Meta-Regression

Stata14.0 is used for estimation in this paper. Table 3 sets out the Logit regression using robust standard errors for all variables included. The Pseudo R2 is 0.189, and the Wald statistic is 24.17 (*p* = 0.0622). Thus, all coefficients (except constant terms) of the model have joint significance statistically. The estimation results in Table 3 show that, in terms of research object features, only subjective support exerts a significant influence, and it more greatly affects the health of the elderly than do social support, objective support, and support availability; this is consistent with the research conclusions of Wei Yan et al. and Chen Changxiang et al. This means that psychological support such as emotional expression and communication is more beneficial to the health of the elderly in rural areas [14,47]. From the perspective of health variables, both health self-assessment and physical health are not significant, indicating no significantly different impacts of social support on the health of the elderly in rural areas with regard to different dimensions of health. This is possibly because social support affects the health of the elderly in rural areas across different dimensions, and different dimensions of health may be correlated with each other.

In terms of social-economic features, the variable “western region” can greatly affect the significance of social support in the health regression coefficient of the elderly in rural areas, indicating that the health of the rural elderly in western regions is more sensitive to social support. This is possibly attributable to richer social support for the rural elderly in western regions than that in central regions. As a result, an equal level of social support exerts a greater “health effect” on the rural elderly in western regions.

In terms of personal traits, social support is more advantageous to significantly improving the health of men over women. This is possibly because social support can bring a much more intense emotional experience to men, thanks to the innate difference in emotions between men and women. Moreover, the phenomenon of “breadwinning men and homemaking women” prevails among the elderly in rural areas. Men are more likely to engage in work outside the home and are more susceptible to the subjective and objective support brought by the social environment [14]. In addition, the impact of social support on the health of the elderly in rural areas is significantly positively correlated with age but not with education level and marital status.

In terms of model data features, the binary model, but not the non-binary model, shows a dramatic negative effect on the significance of social support and the health regression coefficient of the elderly, further supporting the assertion of Cameron and Trivedi [63]. When compared with survey data of other subjects, data from open databases such as CHRLS also greatly affect the significance of social support and the health regression coefficient of the elderly. This is possibly because national open data do not reflect the information contained in regional data and also cover up the differences among the regions’ survey data.

In terms of publication features, neither of the two variables, i.e., neither published year nor journal, significantly affects the significance of social support in the health regression coefficient of the elderly. This means that time difference is not the cause of inconsistent research results, and in other words, the impact of social support on the health status of the elderly has not changed over time. The low impact of the specific journal shows that studies from either published journals or “gray literature” such as academic papers do not affect the significance of research results, proving the nonexistence of publication bias in literature on the impact of social support on the health of the elderly [28].

Based on the above model, the meta-analysis database was used to predict the significance of social support on the health of the elderly in rural areas in a single sample and match it with the actual values. The final prediction accuracy of the Logit model was up to 80.12% (as shown in Table 3).

## 5. Discussion

We found that a diversity of factors affects the significance of the impact of social support on the health of the elderly in rural China and further cause the heterogeneity of relevant research results. We gathered empirical studies on the health and social support of the elderly from the literature, and in the context of controversial conclusions, built a meta-regression database based on 65 articles and explored the causes of inconsistent research results. As a result, the following discussions were drawn: (1) In terms of research object features, subjective support can more greatly affect the health of the elderly than objective support and other dimensions of social support. (2) In terms of social–economic features, the health of the elderly in rural areas in samples involving western regions is more sensitive to social support than that in samples not involving western regions, and social support can more significantly improve the health status of older male elderly than that of younger female elderly. (3) In terms of model data features, the binary model, but not the non-binary model, exerts a significant negative effect on regression of the relationship between social support and the health of the elderly in rural areas; compared with non-open data, open data more adversely affect the relationship between social support and the health of elderly in rural areas. (4) In terms of publication features, neither the published year nor the journal greatly affects the significance of the impact of social support on the health of the elderly in rural areas.

## 6. Conclusions

According to the foregoing research results, we conclude the following: (1) Subjective support rather than objective support positively affects the health of the elderly in rural areas, which conforms to the realities of the accelerating urbanization process, increasing numbers of “left-behind” rural elderly people, and spiritual loneliness. In other words, the left-behind rural elderly have greater spiritual demand than material demand. Therefore, giving more emotional support and spiritual care to the elderly is an effective way to improve their health, and frequent visits by children working in other areas can give the elderly a sense of being cared for and respected and developed self-worth, thus forming a psychological comfort. However, the medical care and social services available for the health of the elderly in rural areas are still far behind those in urban areas in China. Objective support from family and society for the elderly in rural areas is worthy of greater attention and improvement, though it is inferior to subjective support for the improvement of the health of the elderly in rural areas. (2) Due to the poorer natural environment and slower economic development, the rural elderly in western regions enjoy fewer social support resources than do those in the central and eastern regions. In the case of the same level of social support, the elderly in western China obtain more “marginal health benefits” brought by social support. It is important to raise support for social public services in western regions, thus reducing the gaps between them [67]. (3) The open data from CHARLS and the survey data of other subjects are significantly different in the research results on the impact of social support on the health of the elderly in rural areas. As data capture is an important link to be considered in empirical research, data should be acquired around the research objectives of different stages to truly reflect the social environment and then be applied on a per-policy basis. (4) The type of model used in this study affects the significance of the regression coefficient between social support and the health of the elderly in rural areas. This means that variable setting and model selection should be viewed as important factors to be carefully considered in future empirical research, and the loss of information should be minimized to raise the fitting degree between the model and data.

## Figures and Tables

**Table 1:** Basic features of literature included meta-regression.

Author	Published Year	Samples Obtained	Source of Data	Dependent Variable	Independent Variable	Significance
Zhang Weidong et al. [6]	1997	1	Other research (Shanghai)	Health self-assessment	Social support	*p* = 0.001
Zhang Wenjuan et al. [12]	2004	8	CLHLS	Mental health, physical health	Objective support	*p* > 0.100
						*p* < 0.100
					Subjective support	*p* < 0.050
						*p* < 0.010
						*p* < 0.001
Chen Lixin et al. [7]	2005	1	Other research (Wuhan)	Mental health	Social support	*p* < 0.010
Song Lu et al. [33]	2008	1	Other research (Anhui Province)	Mental health	Objective support	*p* = 0.010
Wei Yan et al. [13]	2010	3	Other research (Shaanxi Province)	Mental health	Subjective support	*p* > 0.100
					Objective support	*p* < 0.050
						*p* < 0.001
Wang Jinlong et al. [34]	2011	2	Other research (Jiaxing City)	Mental health	Subjective support	*t* = −3.320
					Support availability	*t* = 2.010
Li Jianxin et al. [17]	2012	3	CLHLS	Health self-assessment, physical health, mental health	Objective support	*p* > 0.100
Gao Yuexia et al. [5]	2013	12	Other research (Nantong city)	Mental health	Social support	*p* = 0.010
						*p* = 0.050
						*p* = 0.100
Jing et al. [35]	2014	2	Other research (Hubei Province)	Physical health, mental health	Objective support	*p* = 0.010
					Subjective support	*p* = 0.001
Tao Yuchun et al. [36]	2014	1	CHARLS	Physical health, mental health	Objective support	*p* = 0.050
Zhou Jing et al. [37]	2016	3	Other research (Anhui Province)	physical health	Objective support	*p* = 0.000
					Subjective support	*p* = 0.010
Bo Ying et al. [25]	2016	7	CHARLS	Health self-assessment, physical health	Objective support	*p* < 0.100
						*p* < 0.010
Shu Fenfen et al. [38]	2017	1	CFPS	Health self-assessment	Subjective support	*p* < 0.050
Zheng Zhidan et al. [16]	2017	1	CHARLS	Health self-assessment	Objective support	*t* = −1.690
Sun Juanjuan et al. [39]	2017	2	CLASS	Mental health	Objective support	*p* < 0.010
Zheng Xiaodong et al. [40]	2017	2	CHARLS	Health self-assessment	Objective support	*p* > 0.100
					Subjective support	*p* < 0.001

Note: Due to limited space, this table only lists a part of the literature published in high-level journals; for all literature included in the meta-analysis database, see Appendix A. The publications by Bo Ying and Wang Jinlong et al. are “gray literature” [26,38]. In this table, CHARLS, CFPS, CLHLS, and CLASS represent China Health and Retirement Longitudinal Study, China Family Panel Studies, Chinese Longitudinal Health Longevity Survey, and China Longitudinal Aging Social Survey, respectively. For the literature samples searched from a single study, the *p* value or *t* value listed as the significance only represents the significance category of the estimation results of multiple samples.

**Table 2:** Variable definition and descriptive statistics.

Variable	Explanation	Mean	Standard Deviation
**Explained variable**			
Significance	Does social support have significant influence on the health of the elderly in rural areas in the research results? Yes = 1, No = 0	0.784	0.413
**Explanatory variables**			
Characteristics of research object			
Social support	Is the support variable a social support? Yes = 1, No = 0	0.240	0.428
Subjective support	Is the support variable a subjective support? Yes = 1, No = 0	0.240	0.428
Objective support	Is the support variable an objective support? Yes = 1, No = 0	0.392	0.490
Support availability	Is the support variable a support availability? Yes = 1, No = 0	0.111	0.315
Health self-assessment	Is the health variable a health self-assessment? Yes = 1, No = 0	0.298	0.459
Physical health	Is the health variable a physical health? Yes = 1, No = 0	0.310	0.464
Mental health	Is the health variable a mental health? Yes = 1, No = 0	0.404	0.492
Socio-economic features			
Household registration	Proportion of respondents with rural household registration	0.679	0.257
Gender	Proportion of male respondents	0.481	0.085
Age	Actual age of respondent	71.921	7.396
Education level	Education level of respondents	6.054	2.603
Marital status	Marriage rate among respondents	0.677	0.169
Western region	Does the sample involve western regions? Yes = 1, No = 0	0.497	0.501
Model data features			
Binary model	Is the model used in regression analysis a binary model? Yes = 1, No = 0	0.351	0.479
Open data	Does the data come from China Health and Retirement Longitudinal Study (CHARLS), China Family Panel Studies (CFPS), Chinese Longitudinal Health Longevity Survey (CLHLS), China Longitudinal Aging Social Survey (CLASS), and other database? Yes = 1, No = 0	0.351	0.479
Publication features			
Year of publication	The publication time of literature, based on 1997	15.444	4.302
Journal literature	Is the literature a published journal paper? Yes = 1, No = 0	0.807	0.396

**Table 3:** Estimation results of meta-regression logit model.

Variable	Nonnormalized Coefficient	Robust Standard Error
**Constant term**	24.057 ***	125.981
**Research object features**		
Social support	0.763	0.621
Subjective support	1.468 **	0.609
Support availability	0.44	0.75
Health self-assessment	0.253	0.48
Physical health	0.45	0.555
**Socio-economic features**		
Household registration	−1.301	1.085
Western region	3.722 ***	1.222
Gender	7.564 **	3.064
Age	0.091 *	0.051
Education level	0.124	0.131
Marital status	1.684	2.284
**Model data features**		
Binary model	−1.022 **	0.497
Open data	−3.450 ***	1.233
**Publication features**		
Year of publication	−0.017	0.062
Journal	−0.255	0.623
Effective samples	171	
Wald chi^2^	24.17	
Pseudo R^2^	0.1886	
Prediction accuracy	80.12%	

***, **, and * represent the significance of model estimation results is less than 0 at the confidence level of 1%, 5%, and 10%, respectively.

## Appendix A

**Table A1:** A summary of the literature included in the study.

Author	Published Year	Samples Obtained	Source of Data	Dependent Variable	Independent Variable	Significance
Zhang Weidong et al.	1997	1	Other investigation (Shanghai)	Health self-assessment	Social support	*p* = 0.001
He Zhaiping	2002	1	Other investigation (Shan Xi Province)	Health self-assessment	Objective support	*t* = 1.534
Liu Zhirong et al.	2003	1	Other investigation (Hefei)	Health self-assessment	Objective support	*p* < 0.001
Liu Zhirong et al.	2003	3	Other investigation (Hefei)	Mental health	Subjective support	*p* = 0.000
					Objective support	
					Support availability	
Zhang Wen juan	2004	8	CLHLS	Mental health, Physical health	Objective support	*p* > 0.100
						*p* < 0.100
						*p* < 0.050
						*p* < 0.010
					Subjective support	*p* < 0.001
Wang Jinying	2004	1	CLHLS	Health self-assessment	Objective support	*p* = 0.382
						*p* = 0.473
Chen Lixin et al.	2005	1	Other investigation (Wuhan)	Mental health	Social support	*p* < 0.010
Song Lu et al.	2006	1	Other investigation (Chaohu)	Mental health	Objective support	*p* = 0.010
Gu Danan et al.	2006	7	CHARLS	Health self-assessment	Support availability	*p* < 0.050
						*p* > 0.100
Song Lu et al.	2006	3	Other investigation (Chaohu)	Health self-assessment	Subjective support	*p* > 0.050
					Objective support	
Tang Dan et al.	2006	3	Other investigation (Beijing)	Mental health	Social support	*p* > 0.050
					Objective support	
Li Jianxin	2007	2	CLHLS	Health self-assessment	Objective support	*p* > 0.100
					Subjective support	*p* < 0.001
Zeng Xianxin	2007	1	CLHLS	Physical health	Social support	*p* < 0.010
Wang Fengling et al.	2008	2	Other investigation (Zhe Jiang Province)	Mental health	Social support	*p* = 0.000
Wang Xumei et al.	2009	2	Other investigation (Wenchuan county)	Mental health	Subjective support	*p* < 0.001
					Support availability	
Wei Yan et al.	2010	3	Other investigation (Shannxi Province)	Mental health	Subjective support	*p* > 0.100
					Objective support	*p* < 0.050
						*p* < 0.001
Jiang Na et al.	2010	4	Other investigation (Yueyang)	Physical health, Mental health	Social support	*p* = 0.000
					Objective support	*p* = 0.002
Wu Jie	2010	3	Other investigation (Tianjin)	Mental health	Subjective support	*p* = 0.000
					Objective support	
					Support availability	
Zhang Jiangying et al.	2010	4	Other investigation (Urumqi)	Health self-assessment, Mental health	Social support	*p* = 0.050
					Subjective support	
					Objective support	
					Support availability	
Zhang Qi et al.	2010	3	Other investigation (Zhe Jiang Province)	Mental health	Subjective support	*p* = 0.001
					Objective support	
					Support availability	
Wang Jinlong et al.	2011	2	Other investigation (Jiaxing)	Mental health	Subjective support	*t* = −3.320
					Support availability	*t* = 2.010
Zuo Dongmei et al.	2011	1	Other investigation (An Hui Province)	Physical health	Objective support	*p* = 0.050
Li Jianxin et al.	2012	3	CLHLS	Health self-assessment, Physical health, Mental health	Objective support	*p* > 0.100
Liu Juanjuan et al.	2012	2	Other investigation (Chaohu)	Health self-assessment	Objective support	*p* = 0.000
Xue Xindong et al.	2012	1	Other investigation (Hu Bei Province and He Nan Province)	Mental health	Support availability	*p* > 0.050
Gao Yuexia et al.	2013	12	Other investigation (Nantong)	Mental health	Social support	*p* = 0.010
						*p* = 0.050
						*p* = 0.100
Song Yueping	2014	6	Other investigation (Eastern)	Health self-assessment, Physical health, Mental health	Subjective support	*p* > 0.100
					Objective support	*p* < 0.100
						*p* < 0.050
Jing et al.	2014	2	Other investigation (Hu Bei Province)	Physical health, Mental health	Objective support	*p* = 0.010
					Subjective support	*p* = 0.001
Tao Yuchun et al.	2014	1	CHARLS	Physical health,	Objective support	*p* = 0.050
				Mental health		
Li Qiang et al.	2014	1	Other investigation (Tianjin)	Mental health	Social support	*p* < 0.010
Chen Changxiang et al.	2014	9	Other investigation (He Bei Province)	Physical health,	Social support	*p* = 0.000
					Objective support	
				Mental health	Subjective support	
Feng Lina et al.	2014	7	Other investigation (He Bei Province)	Physical health, Mental health	Social support	*p* = 0.030
					Objective support	*p* = 0.015
						*p* = 0.049
					Subjective support	*p* = 0.004
						*p* = 0.000
Gu Yanxiang	2014	1	Other investigation (He Bei Province)	Health self-assessment	Social support	*p* = 0.041
Li Shuxing et al.	2014	6	Other investigation (He Bei Province)	Physical health, Mental health	Subjective support	*p* = 0.000
					Objective support	*p* = 0.008
					Support availability	*p* = 0.063
						*p* = 0.245
Liu Xiaoting	2014	4	Other investigation (Zhe Jiang Province)	Health self-assessment, Physical health, Mental health	Social support	*p* = 0.050
						*p* = 0.010
Shi Fanfan et al.	2014	1	Other investigation (Chengdu)	Mental health	Support availability	*p* = 0.036
Xie Pengcheng et al.	2014	1	Other investigation (Nanjing)	Mental health	Objective support	*p* > 0.050
Cheng Xianying et al.	2015	3	Other investigation (He Bei Province)	Mental health	Social support	*p* = 0.000
Hao Xiaoning et al.	2015	2	Other investigation (Beijing)	Health self-assessment	Objective support	*p* < 0.001
					Subjective support	
Ji Bing	2015	1	CHARLS	Health self-assessment	Social support	*p* = 0.000
Li Jing et al.	2015	3	Other investigation (He Bei Province)	Health self-assessment	Social support	*p* = 0.000
Liu Xiguo	2015	1	CHARLS	Health self-assessment	Support availability	*p* = 0.150
Shen Yu	2015	4	CHARLS	Mental health	Subjective support Objective support	*p* > 0.050
Tian Miaomiao et al.	2015	2	Other investigation (He Bei province)	Mental health	Social support	*p* < 0.010
					Subjective support	
Wang Ruimei et al.	2016	1	Other investigation (Shan Dong Province)	Mental health	Subjective support	*t* = −5.410
Zhou Jing et al.	2016	3	Other investigation (Chaohu)	Physical health	Objective support	*p* = 0.000
					Subjective support	*p* = 0.010
Bo Ying	2016	4	CHARLS	Health self-assessment, Physical health	Objective support	*p* < 0.100
						*p* < 0.010
Li Wei et al.	2016	1	Other investigation (Hangzhou)	Health self-assessment	Objective support	*p* = 0.248
Luo Sheng et al.	2016	1	Other investigation (Shan Dong Province)	Mental health	Social support	*p* > 0.050
Song Fengning et al.	2016	1	Other investigation (Nanyang)	Health self-assessment	Subjective support	*p* = 0.150
Shu Panpan et al.	2017	1	CFPS	Health self-assessment	Subjective support	*p* < 0.050
Zheng Zhidan et al.	2017	1	CHARLS	Health self-assessment	Objective support	*t* = −1.690
Sun Juanjuan et al.	2017	2	CLASS	Mental health	Objective support	*p* < 0.010
Zheng Xiaodong et al.	2017	2	CHARLS	Health self-assessment	Objective support	*p* > 0.100
					Subjective support	*p* < 0.001
Sun Jinming	2017	1	CLASS	Mental health	Subjective support	*p* < 0.001
Cheng Shaowen et al.	2017	2	CHARLS	Mental health	Objective support	*p* = 0.050
Bo Ying	2017	7	CHARLS	Health self-assessment	Objective support	*p* = 0.000
						*p* = 0.003
						*p* > 0.100
Dong Haina et al.	2017	1	Other investigation (Zhe Jiang Province)	Health self-assessment	Social support	*p* = 0.549
Liu Xing	2017	3	CHARLS	Mental health	Objective support	*p* > 0.100
					Subjective support	*p* < 0.050
Luo Huiqiang et al.	2017	1	CHARLS	Mental health	Subjective support	*p* > 0.050
					Objective support	
Wang Ping et al.	2017	1	Other investigation (Chaohu)	Mental health	Objective support	*p* > 0.050
Zhang Yi	2017	3	CHARLS	Mental health	Objective support	*p* > 0.050
Mao et al.	2018	1	Other investigation (Chaohu)	Health self-assessment	Objective support	*p* > 0.100
Zhang et al.	2018	1	Other investigation (Shan Dong Province)	Physical health	Objective support	*p* < 0.100
Weng Feiyan	2018	3	CLHLS	Health self-assessment	Subjective support	*p* > 0.050
					Objective support	
